# Adoption of telemedicine: from pilot stage to routine delivery

**DOI:** 10.1186/1472-6947-12-1

**Published:** 2012-01-04

**Authors:** Paolo Zanaboni, Richard Wootton

**Affiliations:** 1 Norwegian Centre for Integrated Care and Telemedicine, University Hospital of North Norway, Tromsø, Norway; 2 Faculty of Medicine, University of Tromsø, Tromsø, Norway

## Abstract

**Background:**

Today there is much debate about why telemedicine has stalled. Teleradiology is the only widespread telemedicine application. Other telemedicine applications appear to be promising candidates for widespread use, but they remain in the early adoption stage. The objective of this debate paper is to achieve a better understanding of the adoption of telemedicine, to assist those trying to move applications from pilot stage to routine delivery.

**Discussion:**

We have investigated the reasons why telemedicine has stalled by focusing on two, high-level topics: 1) the process of adoption of telemedicine in comparison with other technologies; and 2) the factors involved in the widespread adoption of telemedicine. For each topic, we have formulated hypotheses. First, the advantages for users are the crucial determinant of the speed of adoption of technology in healthcare. Second, the adoption of telemedicine is similar to that of other health technologies and follows an S-shaped logistic growth curve. Third, evidence of cost-effectiveness is a necessary but not sufficient condition for the widespread adoption of telemedicine. Fourth, personal incentives for the health professionals involved in service provision are needed before the widespread adoption of telemedicine will occur.

**Summary:**

The widespread adoption of telemedicine is a major -- and still underdeveloped -- challenge that needs to be strengthened through new research directions. We have formulated four hypotheses, which are all susceptible to experimental verification. In particular, we believe that data about the adoption of telemedicine should be collected from applications implemented on a large-scale, to test the assumption that the adoption of telemedicine follows an S-shaped growth curve. This will lead to a better understanding of the process, which will in turn accelerate the adoption of new telemedicine applications in future. Research is also required to identify suitable financial and professional incentives for potential telemedicine users and understand their importance for widespread adoption.

## Background

The sustainability of healthcare systems is a matter for continuing concern [1]. Telemedicine technologies have been proven to work, and are considered to be a viable option [2] in future healthcare delivery, allowing healthcare organisations to provide care in a more economic and comprehensive way. Thus telemedicine is said to be ready for wider adoption [2]. However, telemedicine has a poor record of implementation and a very patchy history of adoption [3], with a slow, uneven and fragmented uptake into the ongoing and routine operations of healthcare [4,5].

Telemedicine became practicable at the end of the 1980s with the availability of low-cost computing and digital telecommunication (e.g. ISDN). Since its inception, many telemedicine applications have been tested in small-scale studies, but most of them have failed to survive beyond the initial (funded) research phase [6], thus not becoming embedded as methods of routine health service delivery.

While successful telemedicine applications certainly exist, they are generally still run by local telemedicine champions and funded on an ad hoc basis. Almost no telemedicine applications have succeeded in reaching large-scale, enterprise-wide adoption [7]. This failure to reach widespread adoption has led to studies of the factors involved in the success and failure of telemedicine applications [8-13].

In telemedicine, success is a relative term, not an absolute attribute. That is, a successful telemedicine application should produce high quality care at low cost in comparison with an alternative such as usual care [14]. Many factors are associated with successful telemedicine applications, including demonstrable savings, adequate financing, acceptance by clinicians, improved access to healthcare and avoidance of travel for patients in rural and remote areas. Successful telemedicine applications must also be sustainable (i.e. they must be adopted into everyday practice and continue to function after any pilot funding runs out, possibly with high activity levels). Fundamentally, a successful application must be cost-effective.

### Widespread implementation of telemedicine applications: the current picture

Over the years a wide range of telemedicine applications has been trialled. Several promising applications seem to be candidates for widespread use in the future, such as telepsychiatry, teledermatology and remote monitoring for diabetes, cardiac and respiratory diseases [15-17]. However, they remain in the early adoption stage.

Teleradiology is the only widespread application that can be considered to have reached full adoption [4]. Teleradiology has become an essential part of the practice of radiology, with broad implications for care delivery and the organisation of work [18]. In 2003, for example, two-thirds of all radiology practices in the US reported using teleradiology, this representing a significant increase from 1999 [19].

There are several reasons for the widespread adoption of teleradiology. First, teleradiology has been demonstrated to provide acceptable diagnostic accuracy in remote reporting. Second, notwithstanding the investments required by the hospitals, teleradiology produces cost savings [20]. Third, in addition to the benefits for physicians and hospital administrators, there are also compelling advantages for patients through avoided travel and rapidity of reporting. Fourth, healthcare payers have set specific reimbursements for teleradiology. Fifth, regulation issues have been addressed (e.g. by the European Society of Radiology) [21]. Finally, teleradiology can benefit from merging with PACS/RIS, thus allowing a shift from shared data to shared workflow [22] and increasing the flexibility of provision, for instance through the use of out-of-hours services.

### Aim of this paper

Because most telemedicine applications are still in the early adoption stage, telemedicine represents an immature technology. There is much debate about why telemedicine has stalled. This is a major -- and still underdeveloped -- challenge in the field of telemedicine, which needs to be strengthened through new research directions.

The objective of this debate paper is to achieve a better understanding of the adoption of telemedicine. In particular, we investigate the reasons why telemedicine has stalled by focusing on two high-level topics: 1) the process of adoption of telemedicine in comparison with other technologies; and 2) the factors involved in the widespread adoption of telemedicine. That is, the topics we have selected are at a high level; clearly there may be other more detailed matters at lower levels, such as specific barriers to adoption like the absence of reimbursement. This debate paper does not attempt to provide a comprehensive and systematic explanation of the adoption of telemedicine. We have made a selection of important topics and formulated certain hypotheses that we believe to be relevant for new research. These hypotheses are all susceptible to experimental verification. We also believe that these hypotheses can assist policy makers and health professionals who are trying to move telemedicine applications from pilot stage to routine delivery.

## Discussion

### Adoption of telemedicine

The term "adoption" refers to the decision of potential users to make full use of an innovation as the best course of action available [23]. An innovation is considered to be fully adopted when the majority of potential users employ it. Before considering the adoption of telemedicine specifically, we discuss the adoption of technology generally, and the adoption of technology in healthcare.

### Adoption of technology generally

**Figure 1:**
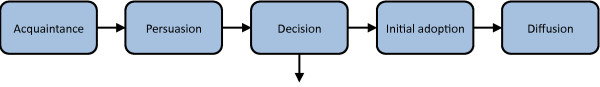
**Stages in the adoption of technology**.

The adoption of technology is the result of a complex decision-making process. It occurs in a number of stages [Figure 1]. In the first stage, an individual or an organisation must become acquainted with the technology under consideration (i.e. unless they know about a technology, they cannot decide to use it). The second stage consists in forming a favourable or unfavourable opinion about the new technology; this is termed persuasion. Here the individual or the organisation wants to know the advantages and disadvantages of the technology. After that, they can decide whether to adopt the technology, or reject it. There is then an initial adoption stage, which may be followed by the widespread implementation of the technology, sometimes termed diffusion. Adoption decisions can be reversed during the diffusion stage, if for example an individual becomes dissatisfied with a technology, or a new or improved technology becomes available [23].

The rate of adoption is the relative speed with which the members of a social system adopt a technology [Additional file 1]. This rate of adoption can be measured through the cumulative percentage of adopters. In practice, adoption is often observed to follow an S-shaped logistic growth curve. In Rogers' classic work on the subject [23], five different kinds of users were identified, based on the time at which they adopted a new technology: 1) innovators, 2) early adopters, 3) early majority, 4) late majority, and 5) laggards.

Rogers identified two important research questions about the process of adoption. The first question is how the early adopters of a technology differ from the later adopters. With this knowledge, late adopters might be identified in advance, and targeted in order to speed up adoption. The second question is how the perceived attributes of a technology affect its rate of adoption. With this knowledge, new technologies could be designed so that they are adopted rapidly.

The above applies to technology adoption generally. How much of this applies to the adoption of technology in healthcare?

### Adoption of technology in healthcare

The adoption of different technologies in healthcare was studied by Russell [24]. She studied the adoption of five technologies which spread widely into US hospitals after 1950. She obtained data from 1953 to 1974 on the uptake of: the post-operative recovery room, the intensive care unit, the respiratory therapy department, diagnostic radioisotope facilities and electroencephalography. There were four main findings: 1) the S-shaped logistic growth curve typically used to describe the process of adoption of innovations in industry also fitted the adoption of these health technologies in US hospitals; 2) the rate of adoption was different for the five technologies; 3) the adoption of a technology started earlier and was faster for larger hospitals; 4) when a technology was attractive, hospitals were as quick to adopt as heavy industry.

If the rate of adoption differs between technologies, what factors mean that one is adopted more quickly than another? Some information on this point comes from a study of the adoption of Computed Tomography (CT) and Magnetic Resonance Imaging (MRI) over the first four years of their availability in the US [25]. The data showed that adoption of both CT and MRI was very rapid; indeed it was so fast that manufacturers were unable to meet the demand initially. This was almost certainly due to the substantial improvement in diagnostic capability and safety compared to existing imaging technologies (i.e. there were major relative advantages of the new imaging techniques in comparison with the technologies available at the time).

However, the rate of adoption of MRI was slower than that of CT. Since MRI became available about a decade after CT, that seems surprising. Why was MRI adopted more slowly? First, MRI did not show an overwhelming relative advantage compared to existing methods of imaging (which by then included CT) at the time it was introduced. Second, MRI was subject to substantial uncertainty due to the technological novelty of the innovation. Third, both technologies were expensive, but the cost of MRI was much higher than the cost of CT. Fourth, governmental regulation was introduced to slow down the adoption of MRI by hospitals [25]. It appears therefore that the adoption of CT and MRI was driven by user demand, and that CT was adopted more quickly due to its major relative advantage.

Governmental regulation may be a factor in adoption, but it appears that it is only a minor factor. This can be seen from a study of the adoption of the automated biochemistry analyzer and the CT scanner among hospitals in New York State [26]. Data for both diagnostic technologies showed that the adoption patterns fitted an S-shaped logistic growth curve. However, the rate of adoption of CT was much higher than that of the automated analyzer. Although CT was more expensive and subjected to more regulation, its adoption was much faster than that of the automated analyzer, which was an unregulated and low-cost technology. We therefore conclude that technological adoption is only weakly influenced by regulatory obstacles. We believe that the crucial determinant of the speed of adoption are the advantages for users.

*H: Advantages for users are the crucial determinant of the speed of adoption of technology in healthcare*.

### Adoption of telemedicine

In the telemedicine literature there is very little quantitative information about the adoption of telemedicine as a method of routine delivery. Examples include the North American telemedicine activities from 1994 to 1999 [27], the email telemedicine network operated by the Swinfen Charitable Trust over the first six years of operation [28], the telemedicine services provided by the Veterans Health Administration in the US [29,30], the telemedicine practice implemented in US prison systems [31], and the teleconsultations administered by the US Department of Defense [32]. However, the value of these telemedicine initiatives is limited to specific organisational settings [29] and it is hard to know how widespread is their use within the organisations concerned, and to draw conclusions about widespread adoption in other public and private healthcare systems. In this respect telemedicine can be considered as a "fact-free zone". As a consequence, we do not know whether telemedicine follows an S-shaped logistic growth curve like other health technologies.

**Figure 2:**
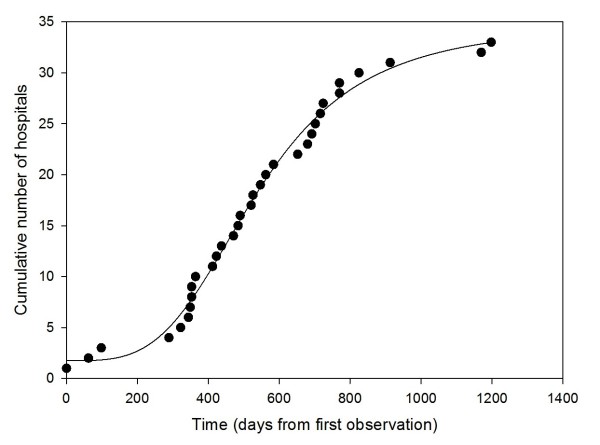
**Adopters of a telemonitoring service for patients with CHF**.

Some interesting data come from a telemonitoring service for patients with chronic heart failure (CHF), which has been widely implemented in the Lombardy Region of Italy, starting in 2006. It is currently in routine use. The implementation of this service was regulated by policy makers, who introduced an experimental regional reimbursement and approved a clinical protocol [33]. This service was offered to all the hospitals in the Region, which could decide to apply for authorization and therefore adopt it on a voluntary basis. The circumstances of adoption are therefore similar to the previous examples concerning CT and MRI in the US. Data were systematically collected from the first introduction of the service. In total, 33 hospitals in the Region used the service over the following four years, starting at different times. Figure 2 shows the growth in the number of service adopters, which seems to follow the S-shaped logistic growth curve typical of health technologies and other innovations.

Thus according to the limited data available, an assumption can be made regarding the similarity between the adoption of telemedicine and that of health technologies generally.

*H: The adoption of telemedicine is similar to that of other health technologies and follows an S-shaped logistic growth curve*.

### Factors in the widespread adoption of telemedicine

The actual adoption of telemedicine is often less than anticipated [34]. Why is this? Innovation theory outlines five attributes that influence the rate of adoption of technologies: 1) relative advantage, 2) compatibility, 3) trialability, 4) observability, and 5) complexity. Relative advantage represents the degree to which a technology is perceived to be better than the existing alternatives [23]. Research shows that relative advantage is the most important factor for the adoption of technology [35,36]. The example above of CT and MRI confirms that a compelling relative advantage leads to rapid adoption.

### Predictors of telemedicine adoption

Perhaps because of the general absence of quantitative data about the adoption of telemedicine, the existence of factors that predict its adoption has been tested mainly through qualitative studies. Various theoretical models have been used. These were originally developed in related fields [37]. For example, the Technology Acceptance Model (TAM) aims to explain user acceptance and to predict the adoption of technologies [38]. In particular, two factors -- Perceived Ease of Use and Perceived Usefulness -- have been identified as important predictors of adoption by users. The TAM has been applied to explain physicians' decisions to accept telemedicine. In a study conducted in public tertiary hospitals in Hong Kong, the TAM provided a reasonable depiction of physicians' intentions to use telemedicine [39].

Other theoretical models have been used to investigate additional factors that might influence the adoption of telemedicine [40], including the Theory of Planned Behaviour (TPB) and the Theory of Interpersonal Behaviour (TIB) [41]. More recently, May and colleagues developed a Normalization Process Theory (NPT) to explain the implementation, embedding and integration of complex health interventions into everyday practice [42]. In the case of telemedicine, qualitative data collected through observation and interviews suggested a number of requirements for its successful adoption. These include: 1) a positive link with a policy level sponsor, 2) successful structural integration, 3) cohesive, cooperative groups, and 4) integration at the level of professional knowledge and practice [43].

Scholars have also focused on several barriers that should be addressed for telemedicine adoption to occur. Reimbursement and legal/regulatory issues are claimed to be the most common barriers explaining the diffusion trends for many telemedicine applications [44]. Whitten and Mackert have pointed out that the provider is the most important initial gatekeeper for the deployment of telemedicine, and that project managers must keep providers' needs (ease of use and incentives) in mind when designing a telemedicine system [45]. Other barriers include technology integration, interoperability, standardization, security, lack of time and financing available [46].

### Evidence in telemedicine -- advantages to society

One factor affecting the widespread adoption of telemedicine can be assumed: the evidence that it is a cost-effective method of practice. Without information on the costs and effectiveness of interventions, decision makers -- and thus adopters -- run the risk of introducing services that are not cost-effective for society [47]. Information about a technology allows the uncertainty about its adoption to be reduced. From a societal perspective, this is an ethical matter, since resources expended on an ineffective service are not available for other, demonstrably effective alternatives.

Systematic reviews have identified evidence for the advantages of telemedicine to society [48,15]. However there are still significant gaps in the evidence base between where telemedicine is used and where its use is supported by high-quality evidence [49]. In a recent systematic review of reviews [17], 21 out of 80 heterogeneous reviews concluded that telemedicine was effective. A recent Cochrane systematic literature review concluded that there is clear evidence of the clinical benefits of telemonitoring for patients with CHF, while more evidence is still required on the cost-effectiveness [50].

It has been claimed that there is no good evidence that telemedicine is a cost-effective means of delivering healthcare [51]. However, there has recently been a considerable increase in economic evaluations in telemedicine [52]. Although few economic evaluations of telemedicine provide reliable information for decision making [53], there is evidence of the cost-effectiveness in certain telemedicine services [54], and decisions can be made on the basis of limited -- but sufficiently detailed -- studies [47].

Evidence is regarded as a requirement for the introduction of a new drug or treatment. Similarly, evidence is needed to evaluate the advantages of telemedicine applications to society and to convince professionals and policy makers about implementation [55]. Although there is evidence of the cost-effectiveness of telemedicine in certain situations, its widespread adoption has not occurred. The main implication is that evidence of cost-effectiveness is a necessary but not sufficient condition for adoption.

*H: Evidence of cost-effectiveness is a necessary but not sufficient condition for the widespread adoption of telemedicine*.

### Personal incentives in telemedicine -- advantages to health professionals

One way of viewing the strict evidence of the cost-effectiveness of telemedicine is to regard this as representing an advantage to society as a whole. However, this is not the same as the advantage to the individual user (e.g. doctor or nurse) who makes a decision to employ telemedicine when managing a patient.

Here it is worth distinguishing between the decision to make telemedicine possible in a healthcare system (i.e. to provide the equipment for doing it) and the decision to employ it in practice. While the first is usually a decision at organisational or governmental level, the latter is normally made by individual health professionals.

The example of telemedicine in Malaysia is relevant here, one of the few countries with specific legislation and guidelines for telemedicine [56]. The Malaysian government studied the evidence for telemedicine from other parts of the world and attempted to implement it across the national healthcare system. Despite an investment of US$5.5 million in a national telemedicine system, health professionals handled only a few hundred cases before the project was withdrawn for re-planning [57]. As the history of telemedicine so clearly shows, governments can provide the technology for telemedicine, but unless health professionals are persuaded, the equipment will not be used.

In a study comparing adopters and non-adopters of telemedicine, the number of telemedicine referrals made by adopters was significantly correlated with adopters' perceptions of the advantages [58]. Health professionals' perceptions, together with organisational and cultural structures affecting health, legal issues, technical difficulties, time, convenience, cost, training and familiarity with the equipment, have been claimed to be facilitators for the adoption of telemedicine [34]. In another study, some differences in attitudes to telemedicine were found between users and non-users. In particular, health professionals who used telemedicine in their work had more positive attitudes towards it [59]. An extensive search of the telemedicine literature claimed that telemedicine is successful, and therefore adopted into routine practice, when it is perceived as a benefit and as a solution to political and medical issues [10]. Moreover, different parties in telemedicine are likely to have very different perspectives, which may influence their decisions about adoption. For example, health professionals at remote sites frequently view telemedicine as having a relative advantage, while those at hub sites often view it as offering no relative advantage and requiring changes to their existing practices and roles [34].

Thus a crucial factor in the adoption of telemedicine is the attitude of the health professionals on the ground. Since most telemedicine applications require additional effort and technical expertise, the use of telemedicine is almost always more time and trouble than practising in the ordinary way. We believe that before health professionals will seriously consider the use of telemedicine, there must be some personal advantage to the user, in addition to the general advantages to society.

*H: Personal incentives for the health professionals involved in service provision are needed for the widespread adoption of telemedicine to occur*.

### What kind of incentives?

The provider is the most important initial gatekeeper for telemedicine, and therefore incentives should be kept in mind when implementing telemedicine applications [45]. What sort of incentives to use telemedicine might be appropriate? They could include both financial incentives and professional incentives [60].

Financial incentives in healthcare may take the form of direct payments to health professionals (e.g. fee-for-service) or indirect payments (e.g. income to spend on clinical activities, flexibility over a cash-limited budget) [60]. A systematic review of the impact of financial incentives for health professionals has shown evidence that these do affect their behaviour [61]. For example, there was a positive response from British General Practitioners (GPs) to financial incentives [62,63]. Moreover, pay for performance policies have been promoted to accelerate improvements in the quality of care [64,65]. Financial incentives have also been considered as important factors in helping communicate the relative advantages of telemedicine to potential adopters [66], thus motivating health professionals to use it [16,67,68].

In addition, professional incentives can be employed in order to influence health professionals. Examples include status, congeniality of work, career progression, client differentiation, clinical profile [60] and public recognition (e.g. report cards) [69,70]. The high initial physician time costs have sometimes been seen as a major barrier to adoption of new technologies [71]. Support for organisational changes to health professionals, including training, educational material and technical support, can help them to carry out a time-consuming workflow more efficiently [72]. Professional incentives have also been investigated in order to understand why the adoption of telemedicine remains low. Training, appropriate personnel [73], support, research ability [16] and knowledge translation involved in frequent remote interactions [74] have been claimed to motivate health professionals to use telemedicine and to speed up its implementation [16].

## Summary

The widespread adoption of telemedicine is a major -- and still underdeveloped -- challenge that needs to be strengthened through new research directions. We have formulated four hypotheses about telemedicine adoption, which are all susceptible to experimental verification.

First, advantages for users are the crucial determinant of the speed of adoption of technology in healthcare. The rapid growth of two major imaging technologies, CT and MRI, shows clearly that health technologies are adopted if users, especially health professionals, want them (i.e. if they perceive that those technologies substantially improve the way they can practice). We thus believe that these considerations should be taken into account in future studies addressing the adoption of telemedicine.

Second, adoption of technology tends to follow an S-shaped logistic growth curve where the adopters decide to use a technology at different times. This also applies to the adoption of new health technologies. We believe that data about the adoption of telemedicine should be collected from applications implemented on a large-scale, to test the assumption that the adoption of telemedicine follows an S-shaped growth curve. This will lead to a better understanding of those who are likely to adopt new telemedicine applications in future.

Third, before the widespread adoption of a telemedicine application can be justified, formal evidence of the advantages to society is required. Although there is evidence of the cost-effectiveness of telemedicine in certain situations, its widespread adoption has not occurred. We thus believe that the evidence of cost-effectiveness is a necessary but not sufficient condition for adoption.

Fourth, evidence is crucial to prove the advantages of telemedicine to society, but advantages to health professionals in the form of personal incentives are also needed for the widespread adoption of telemedicine to occur. Governments can provide health professionals with the technology, but the majority of potential users need to perceive compelling relative advantages of telemedicine over existing practices in order to adopt it. We believe that research is also required to identify suitable financial and professional incentives for potential telemedicine users and understand their importance for widespread adoption.

## Competing interests

The authors declare that they have no competing interests.

## Authors' contributions

Both authors contributed directly to the planning, execution and analysis of the work reported. Both authors read and approved the final manuscript.

## Pre-publication history

The pre-publication history for this paper can be accessed here:

http://www.biomedcentral.com/1472-6947/12/1/prepub

## Supplementary Material

Additional file 1 **Rate of adoption**. Description of the S-shaped growth curve characterizing the rate of adoption of technology. Click here for file
