# Supplementary material for: Adoption of telemedicine: from pilot stage to routine delivery
Source: BMC Med Inform Decis Mak. 2012 Jan 4;12:1. doi: 10.1186/1472-6947-12-1 (PMC3280930; doi:10.1186/1472-6947-12-1)
Supplement: Additional file 1 — Rate of adoption. Description of the S-shaped growth curve characterizing the rate of adoption of technology. [file 1472-6947-12-1-S1.PDF]

Rate of adoption.

The S-shaped growth curve that is commonly used to describe adoption is

$$P = K / [1 + exp-(a + b.t)]$$

where

P is the proportion of users who have adopted the technology at time t
K is the ultimate proportion of users who will have adopted it when the process is complete
a is a constant (the time at which adoption begins)
b is a constant the rate of adoption)

If both sides of the equation are divided by (K - P), then after taking logs

$$ln[P / (K - P)] = a + b.t$$

That is, the log of the ratio of the number adopting to the number not adopting is a linear function of time. The coefficient *b* is the rate at which this log ratio changes with time, i.e. it is a measure of the speed of adoption.

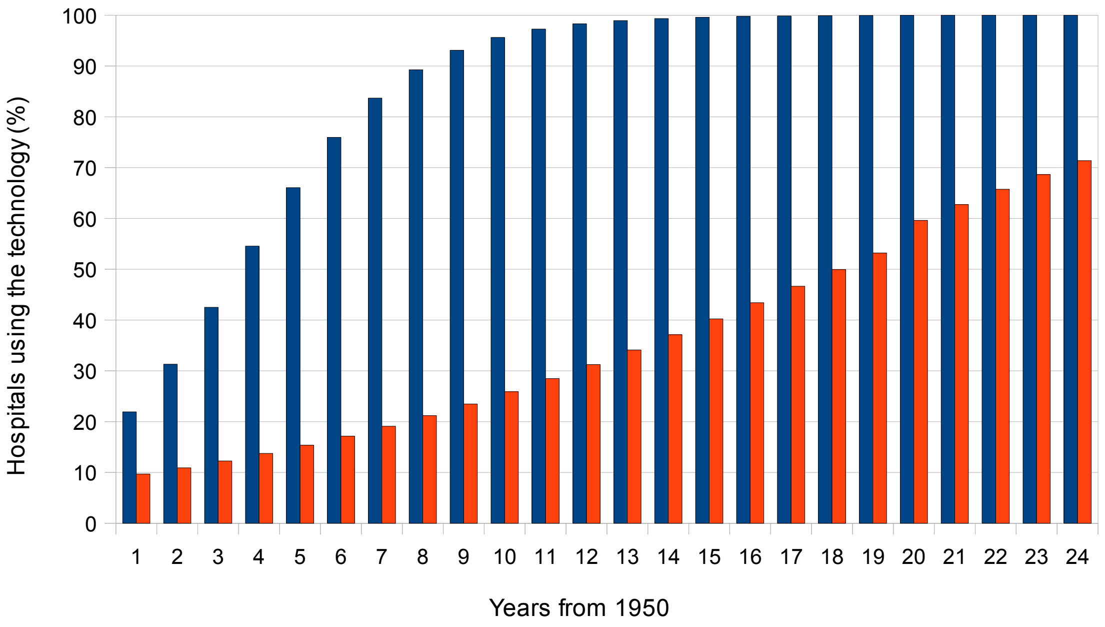

Blue curve: early adoption and rapid diffusion
Red curve: later adoption and slower diffusion, i.e. smaller *a* and smaller *b*
